# The muscle development transcriptome landscape of ovariectomized goat

**DOI:** 10.1098/rsos.171415

**Published:** 2017-12-20

**Authors:** Sihuan Zhang, Han Xu, Xinfeng Liu, Qing Yang, Chuanying Pan, Chuzhao Lei, Ruihua Dang, Hong Chen, Xianyong Lan

**Affiliations:** College of Animal Science and Technology, Northwest A&F University, Yangling, 712100, Shaanxi, People's Republic of China

**Keywords:** transcriptome, ovariectomy, goats, muscle, development, growth

## Abstract

In practical production, almost all rams and about 50% of ewes are used to fatten. Researchers have proved that ewe ovariectomy could improve the productivity significantly, but the specific molecular mechanism is still unknown. In this study, five independent cDNA libraries (three and two from ovariectomized and normal ewe longissimus dorsi samples, respectively) were constructed to thoroughly explore the global transcriptome, further to reveal how the ovariectomized ewes influence muscle development by Illumina2000 sequencing technology. As a result, 205 358 transcripts and 118 264 unigenes were generated. 15 490 simple sequence repeats (SSRs) were revealed and divided into six types, and the short repeat sequence SSR (monomers, dimers, trimers) was the domain type. Single nucleotide polymorphism analysis found that the number of transition was greater than the number of transversion among the five libraries. Furthermore, 1612 differently expressed genes (DEGs) (Log2fold_change > 1 and *p *< 0.05) were revealed between ovariectomized and normal ewe groups, in which 903 genes were expressed commonly in the two groups, and 288 and 421 genes were uniquely expressed in normal and ovariectomized ewe groups, respectively. Gene Ontology (GO) analysis categorized all unigenes into 555 GO terms and 56 DEGs were significantly categorized into 43 GO terms (*p *< 0.05). KEGG enrichment analysis annotated 12 976 genes (containing 137 DEGs) to 86 pathways, among them 24 and 11 DEGs involved in development and reproduction associated pathways, respectively. To validate the reliability of the RNA-seq analysis, 22 candidate DEGs were randomly selected to perform quantitative real-time polymerase chain reaction. The result showed that 9 and 1 genes were significantly and approximately significantly expressed in control and treatment group, respectively, and the results of RNA-seq are believable in this study. Overall, these results were helpful for elucidating the molecular mechanism of muscle development of ovariectomized animals and the application of female ovariectomy in fattening.

## 1. Introduction

With the improvement of living standards and the demand for mutton, in practical production, as much as 50% of ewes still are used to fatten. Ram castration has been applied in production and achieved effective results [1,2]. Many researchers have proved that ewe ovariectomy (removal of the ovary) could improve the productivity significantly like ram castration. Study on mice found that the ovariectomized groups showed a greater body mass than the group without ovariectomy, no matter fed on standard chow or high-fat diet [3]. Studies on goat found that meat performance and muscle tissue nutrient content of the ewe kid could be improved by ovariectomy [4,5]. Zhang *et al.* slaughtered 18 goats (nine goats in ovariectomy group and nine goats in control group) on day 50 after ovariectomization, and measured meat performance. They found that the average daily gain, live weight, carcass weight, net meat mass, and loin eye area of ovariectomized goats were significantly higher than those of the control goats (*p *< 0.05). Meanwhile, an average loss of bone weight (0.40 kg) was observed in the ovariectomized goats compared with the unovariectomized goats (*p *< 0.05). But there was no significant difference between the ovariectomized group and control group in fat weight (*p *> 0.05) [5]. Although these findings proved that ewe ovariectomy could improve the productivity significantly, the specific molecular mechanism is still unknown. Thus it is meaningful to reveal the molecular mechanism of ewe ovariectomy improving the productivity and to applied ovariectomy in practice production.

Ovariectomy has been used as effective disease treatment method and breeding control in animals, such as pig, dog, rabbit, rat, even hippopotamus [6,7]. Moreover, experimental studies often apply ovariectomized animals to mimic menopause, because the characters of ovariectomized animals resemble the metabolic alterations observed in women [8,9]. Moreover, ovariectomized animals serving as disease or medicine research models are widely adopted [10,11]. Ovariectomy-induced osteoporosis rabbit models have been used to examine the effects of autologous dedifferentiated fat cell transplantation on bone regeneration and investigate the effects of a traditional Chinese herb product, OsteoKing, for the treatment of bone disease [12,13]. Ovariectomized rat was chosen as the model to analyse the influence of ovariectomy on alveolar bone and tibiae [14]. Thus ovariectomy is a mature technique, which can be applied to goat or other livestock in practical production.

Boer goat is a world famous meat-type breed, which has great reproductive capacity and growth rate. Also, Boer goat is one of the most adaptable breeds to fit the natural ecological environment and it has been spread to many countries [15–17]. Hybridization experiment showed that Boer goat could improve the production performance of local goat breed [18]. Guanzhong dairy goat is a well-known Chinese dairy goat breed, which is widely distributed in the Guanzhong region in Shaanxi Province of China [19]. The introduction, breeding, and hybridization with Guanzhong dairy goat of Boer goat have strongly promoted the development of mutton goat industry in China. The hybridization test of Boer goat and Guanzhong dairy goat started in 1995 in Shaanxi province, China. Early practical test results showed that the F1 hybrid of Boer goat and Guanzhong dairy goat has better growth rate than the F1 hybrid of Boer goat and other goat breeds. And the F1 hybrid of Boer goat and Guanzhong dairy goat could well adapt to the local ecology characteristic of Shaanxi province. Nowadays, the F1 hybrid of Boer goat and Guanzhong dairy goat is used for fattening in production practice.

Muscle is one of the main factors which influence the growth and development of animals. Longissimus dorsi being muscle tissue, its growth rate is relatively stable compared to the other muscles on other body parts, such as leg muscle. The growth rate of longissimus dorsi is affected by the growth characteristic of goat itself and the raising level, but is less affected by exercise intensity. There are some high-throughput sequencing of RNA (RNA-seq) studies that pay attention to the growth and development of animals just detecting the mRNA or miRNA expression levels in longissimus dorsi muscle [20,21]. According to the results, this study conjectured that ovariectomy might increase the mRNA expression levels of some genes which promote muscle growth and development.

RNA-seq is an efficient way to map and quantify transcriptome and to analyse global gene expression changes in different samples [22–24]. Thus this study regarded F1 hybrid of Boer goat and Guanzhong dairy goat as model animal to thoroughly investigate the global transcriptome of ovariectomized ewe and normal ewe longissimus dorsi muscle samples by RNA-seq method. The objective of this study was to clarify the molecular mechanism of ovariectomy affecting animal growth, offer valuable resource for female animal fattening and provide essential information for further research on muscle development of ovariectomized animal.

## 2. Material and methods

### 2.1. Experimental design and sample collection

This study used six female Boer hybrid goats (Boer goat♂ × Guanzhong dairy goat ♀) as models, which were chosen from national mutton industrial technology system Baoji comprehensive experimental station, Shaanxi Province, China. Six female hybrid kids with similar body weight at about five months old were randomly divided into treatment group (three kids) and control group (three kids). The goats in the treatment group were ovariectomized at the beginning of the experiment and the goats in control group were untreated. All the six goats were slaughtered at the 50th day. The longissimus dorsi muscle samples were collected immediately after slaughtering, and were snap-frozen in liquid nitrogen, then stored at −80°C until RNA extraction [25].

### 2.2. RNA extraction, library construction and sequencing

Total RNA was extracted from the six samples using Trizol reagent (Takara, Dalian, China) according to the manufacturer's instructions. The RNA purity was determined via the OD_260_/OD_280_ ratio and OD_260_/OD_230_ ratio. The integrity was evaluated using gel electrophoresis and checked by RNA integrity number (RIN) value. RNA samples with a RIN value greater than 8.0 (8.0 out of 10.0), OD_260_/OD_280_ ratio greater than 1.9, OD_260_/OD_230_ ratio no less than 1.7 and the amount of RNA no less than 20 µg were selected for deep sequencing [26].

The RNA was treated with DNase I for 30 min at 37°C to remove residual DNA. Then mRNA was enriched and purified using beads with oligo (dT) [27]. The purified mRNA was fragmented to approximately 350 nt fragments using the RNA fragmentation kit. The first strand cDNA was synthesized using purified RNA fragments as template and hexamer primers. After the first strand was synthesized, the buffer (Invitrogen, 20 µl), dNTPs (0.25 mM/μl), RNaseH (0.05 U/μl) and DNA polymerase I (0.5 U/μl) were added to synthesize the second strand cDNA [26]. The short cDNA fragments were further purified using the QIAQuick PCR extraction kit (LianChuan Sciences, Hangzhou, China). The samples were then subjected to 3′ end repaired, a single ‘A’ base and an adapter ligation. Agarose gel electrophoresis was used to filter the suitably sized fragments (350 ± 50 bp), which would be used as the templates for the polymerase chain reaction (PCR) amplification and RNA-seq library construction. Then the sequencing of the libraries was performed using Illumina HiSeq 2000 (LianChuan Sciences, Hangzhou, China).

Taken together, there was one sample that did not meet the requirement. Thus finally we constructed five libraries to investigate the global transcriptome of ovariectomized ewes (treatment group: three samples) and normal ewes (control group: two samples) longissimus dorsi muscle samples by deep RNA sequencing method.

### 2.3. Transcript and unigene assembly and annotation

The valid data, which can be used for subsequent analysis, were obtained by removing the adapters and low quality reads in raw reads. The transcript and unigene assembly was carried out using short assembly program Trinity method [28,29]. The assembled datasets were deposited in a publicly available database: Gene Expression Omnibus (GEO): GSE84110: http://www.ncbi.nlm.nih.gov/geo/query/acc.cgi?acc=GSE84110.

All assembled unigenes were compared with public database, NCBI non-redundant protein sequences (NR), SWISS-PROTPROT, Kyoto Encyclopedia of Genes and Genomes (KEGG), euKaryotic Ortholog Groups (KOG) and Pfam to make functional annotation by similarity analysis. Sequences alignment using BLAST software, only a significance threshold of *E*-value < 10^−5^ can be used as annotation information.

### 2.4. Identification of unigene simple sequence repeat and single nucleotide polymorphism

Simple sequence repeat (SSR), also named microsatellite DNA, is composed of many repetitive nucleotide sequences. The numbers of repetitive nucleotide sequences in different individuals result in polymorphism in group. The SSR in this study was detected by MiscroSAtellite software using assembled unigenes as reference sequences. Single nucleotide polymorphism (SNP) is similar to SSR, which can be used for genetic diversity analysis, marker-assistant selection, QTL analysis, genetic linkage map construction etc. This study analysed the SNPs in gene coding sequences using Bowtie software, and screened reliable SNP loci using Samtools software.

### 2.5. Differently expressed genes analysis

All reads were mapped onto the non-redundant set of transcripts to quantify the abundance of assembled transcripts. The gene expression level was computed by Bowtie 0.12.8 using single-end mapping method, and further measured by the reads per kilobase of exon model per million mapped reads value (RPKM), which can be calculated as follows: *RPKM *= total exon reads/[mapped read (millions) * exon length (KB)] [30]. The high RPKM represents the high expression level of gene. The Log2fold_change (calculated by log2 (sample_2 RPKM/sample_1 RPKM)) and *P* value were used to calculate the different expression between two samples. False discovery rate (FDR) is correct for the *p* value [31]. When *p* < 0.05, higher values of Log2fold_change and lower FDR showed more significant differently expressed genes (DEGs). Moreover, the DEGs were subjected to Gene Ontology (GO) and KEGG analysis.

GO database, an international standard gene functional classification system, was used to predict and illuminate the function of gene product on molecular, biological process and cellular component [32]. The DEGs were mapped to the GO terms in the database (http://www.geneontology.org/) firstly. Then the gene numbers in every GO term were calculated and the significantly enriched GO terms were found using corrected *p* ≤ 0.05 as a threshold. The *p* value was calculated by the following formula:

$$p=1-\sum_{i=0}^{m-1}\frac{\left(\begin{array}{c} M\\ i \end{array}\right)\left(\begin{array}{c} N-M\\ m-i \end{array}\right)}{\left(\begin{array}{c} N\\ n \end{array}\right)},$$

where *N* is the number of all genes; *n* is the number of the DEGs in *N*; *M* represents the number of the genes which were categorized to a certain GO term; *m* represents the number of the DEGs which were categorized to a certain GO term.

KEGG (http://www.genome.jp/kegg/) was used to perform a pathway enrichment analysis of unigenes [33]. Pathway enrichment analysis can confirm the main biochemical pathway and signal pathway which DEGs participated in. The significantly enriched KEGG pathway using corrected *p *≤ 0.05 as a threshold. The calculation formula of the *p* value was the same as that in the GO analysis, where *N* is the number of all genes with pathway annotated; *n* is the number of the DEGs in *N*; *M* is the number of the genes annotated to a certain pathway; *m* is the number of the DEGs annotated to a certain pathway.

### 2.6. Experiment verification and statistical analysis

To quantitatively determine the reliability of our transcriptome data, 22 significantly DEGs were selected randomly from different pathways and functional categories to test their expression levels via the quantitative real-time PCR (qRT-PCR) method. Ribosomal protein L19 (*RPL19*), which is the most stable housekeeping gene in goat muscle samples among *PITX1*, *PITX2*, *GAPDH* and *beta-actin* (unpublished data), was used to normalize the expression of target genes in longissimus dorsi muscle samples. Total RNA samples were reversed to cDNA using the PrimeSript™ RT reagent kit (TaKaRa, Dalian, China) according to the manufacturer's recommendations. qRT-PCR was performed using the SYBR^®^ Premix Ex Taq™ kit (TaKaRa, Dalian, China) in a Bio-Rad CFX96 real-time PCR system (Hercules, CA, USA). The primers used in this study are shown in electronic supplementary material, table S1. The qRT-PCR reaction system composition and program are the same as in a previous study [34]. Individual samples were run in triplicate.

Relative expression of mRNA was calculated using the 2^−ΔΔCt^ method [35]. Student *t*-test was used to analyse independent samples via SPSS (version 17.0, SPSS, Inc., Chicago, IL, USA), and data were expressed as the mean ± 1 standard deviation of duplicates. A *p* value < 0.05 was considered statistically significant.

## 3. Results and discussion

### 3.1. De novo assembly and functional annotation

Five libraries were constructed to obtain a global view of the transcriptome of normal ewes (control group: BZJ_1_N and BZJ_3_N) and ovariectomized ewes (treatment group: BZJ_2_T, BZJ_4_T and BZJ_5_T) longissimus dorsi muscle samples by deep RNA sequencing method. As a result, we acquired an average 5.3 billion clean reads from each library after filtering the redundant sequences. The lowest valid ratio (reads) is as high as 99.81% (electronic supplementary material, table S2).

**Figure 1.**
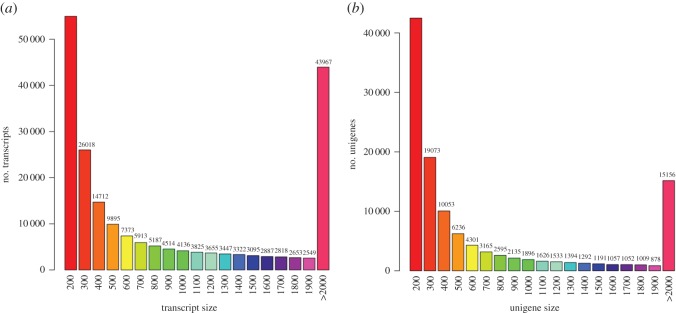
The length distribution of transcript (*a*) and unigene (*b*). The horizontal axis shows the length extent of transcript and unigene, and the vertical scale shows the numbers of transcript and unigene in the sequence data.

**Figure 2.**
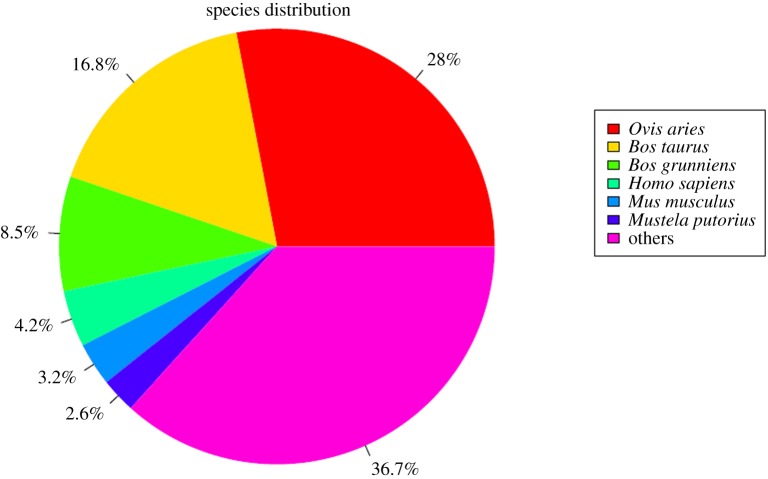
Unigenes distribution in NCBI non-redundant protein sequences database.

Since the goat genome is not comprehensive enough, we used de novo assembly method to construct transcripts and unigenes from RNA-seq reads by Trinity [28]. The high quality reads (*Q* > 20) were assembled into transcripts and unigenes, with a fixed k-mer of 25 [36,37]. In total, 205 358 transcripts and 118 264 unigenes were generated, with average lengths of 1291 bp and 919 bp, respectively (figure 1). All assembled unigenes were firstly aligned to NR protein database, which contains the protein sequences translated by all DNA sequences in GenBank and come from other databases. According to the results of blast, 44 504 (37.63%) unigenes obtained annotations in NR database, and they were similar to genes in other species, such as *Ovis aries* (28%), *Bos taurus* (16.8%) and *Bos grunniens* (8.5%) (figure 2). To better understand the functionality of these unigenes, they were further blast in other databases. The annotation numbers and ratios of unigenes against various databases were SWISS-PROTPROT 41 335 (34.95%), KEGG 39 389 (33.31%), KOG 38 284 (32.37%) and Pfam 31 321 (26.48%). The rate of the annotated genes in this study was the same as that in another study of goat [38].

### 3.2. The distribution of unigene simple sequence repeat and single nucleotide polymorphism

**Figure 3.**
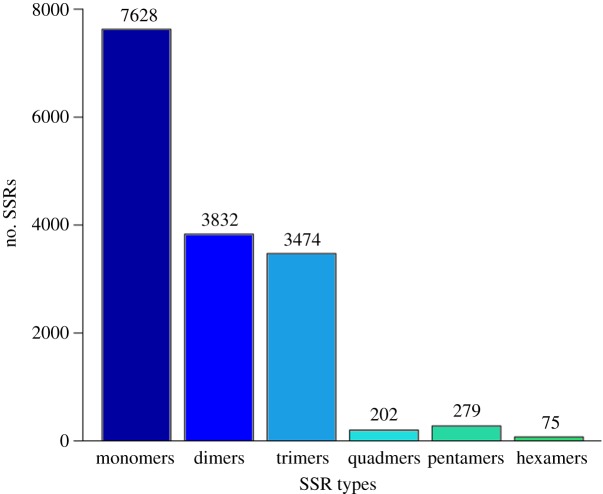
The SSR numbers of each SSR type.

**Figure 4.**
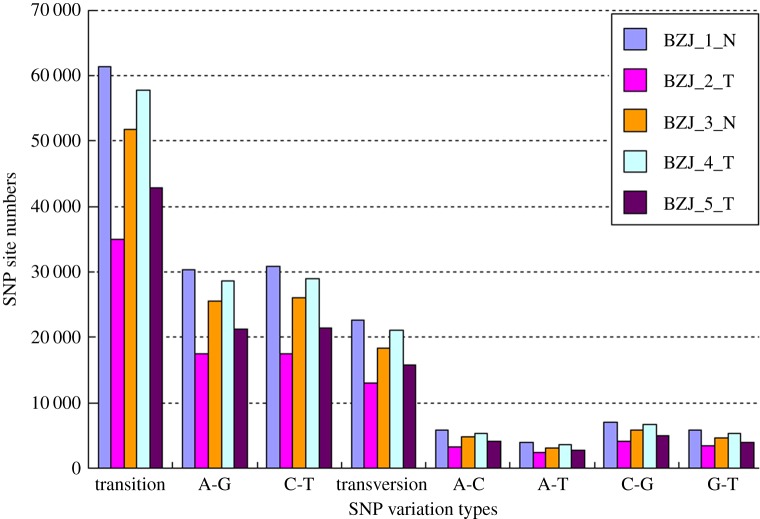
The summary of the numbers of different single nucleotide polymorphism (SNP).

After SSR analysis, a total of 15 490 SSRs were revealed, and they were divided into six types according to the length of the repeat sequence. The numbers for each type were monomers (7628), dimers (3832), trimers (3474), quadmers (202), pentamers (279) and hexamers (75) (figure 3). Among these, the short repeat sequence SSR (repeat nucleotide ≤ 3) was the domain type. The SNP analysis found 84 055 (BZJ_1_N), 84 055 (BZJ_2_T), 70 044 (BZJ_3_N), 70 044 (BZJ_4_T) and 58 575 (BZJ_5_T) SNPs in the transcriptome alignment file of the five samples. The transitions were more than the transversions among the five libraries. The numbers of different SNP types in the five libraries are shown in figure 4. SSR and SNP molecular markers are the basis for genetic mapping and comparative genomic analysis. Although some SSR markers have been researched in goat, few studies have investigated the SSRs on mRNA [19]. The distribution of the SSRs and SNPs revealed in this study in the genome was consistent with other studies in goat [38]. The results confirmed that RNA-seq is an efficient method to uncover genetic variations in transcribed regions [39].

### 3.3. Differentially expressed genes analysis

**Figure 5.**
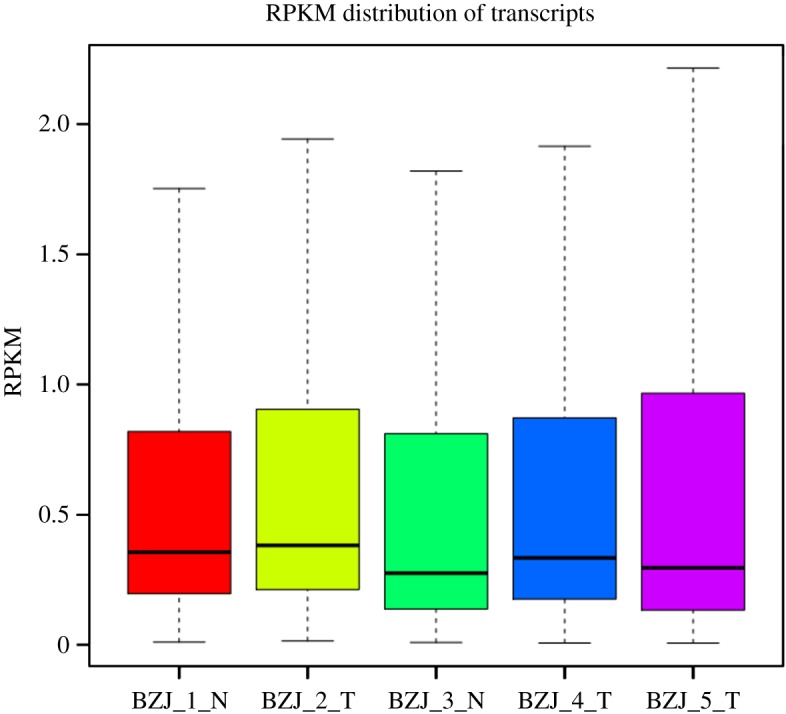
Expression levels of the five samples. The vertical scale shows the RPKM values of each sample. Every box region represents five statistics, which, from top to bottom are, in order, maximum, 3rd quartile, median, 1st quartile and minimum.

**Figure 6.**
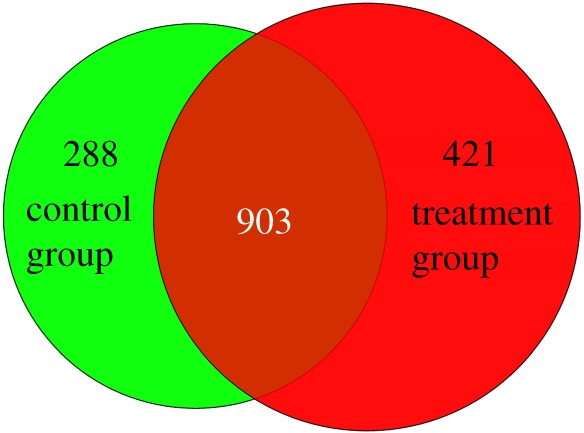
The differentially expressed genes that are uniquely or commonly expressed in control group (normal ewes) and treatment group (ovariectomized ewes) longissimus dorsi muscle samples. The numbers in each section indicate the number of differentially expressed genes.

**Figure 7.**
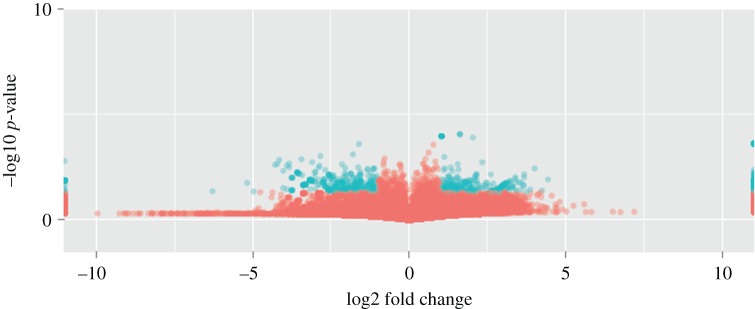
Volcano plots of the differently expressed genes in control group (normal ewes) and treatment group (ovariectomized ewes) longissimus dorsi muscle samples. The blue plots represent the significance; the red plots represent no significance; log2 fold change is equal to log2 (treatment group value/control group value).

The transcript expression levels of the five samples were calculated by RPKM values and exhibited using box plot (figure 5). Furthermore, a total of 1612 (Log2fold_change > 1 and *p *< 0.05) and 216 (Log2fold_change > 1 and *p* < 0.01) DEGs were revealed between control group and treatment group longissimus dorsi muscle samples (electronic supplementary material, table S3). In 1612 DEGs, 718 genes were upregulated and 894 gene were downregulated in longissimus dorsi muscle samples of ovariectomized ewes. Meantime, 903 genes were expressed commonly in the two groups, 288 and 421 genes were uniquely expressed in control and treatment group, respectively (figure 6). Volcano plots could infer the whole pattern of the DEGs and determine any systematic bias that may be present between conditions from the levels of log2 fold change and −log10 *p* value. Log2 fold change represented the change of genes expression multiple between samples. And −log10 *p* value represented the statistically significant DEGs between samples. Thus we used the volcano plots to explore the relationship between the fold change and the significance (figure 7).

**Figure 8.**
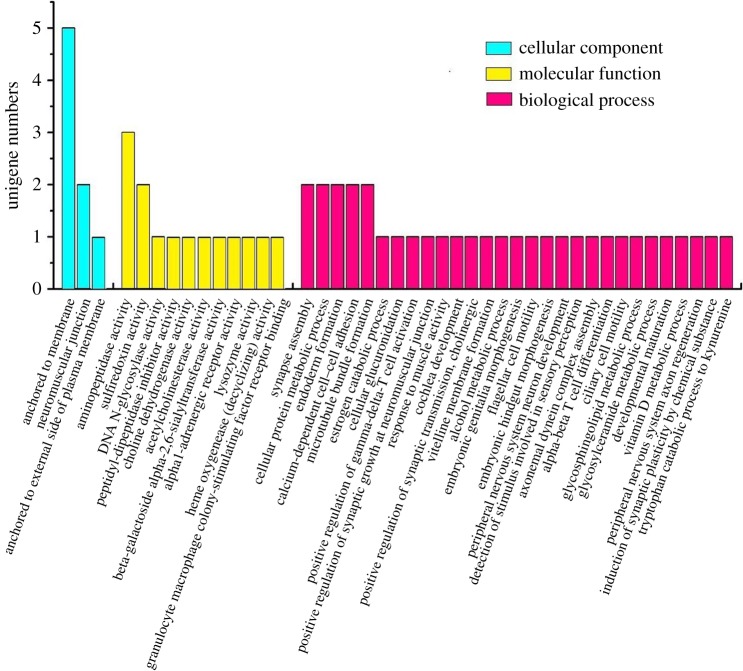
Significantly enriched GO terms of differently expressed genes in the analysed goat longissimus dorsi muscle samples.

To investigate the potential function of the DEGs, GO analysis was performed to categorize DEGs into different biological function class. All unigenes (including 1612 DEGs) were categorized into 555 GO terms in the GO database. But only 56 DEGs were significantly categorized into 43 GO terms (*p *< 0.05) (electronic supplementary material, table S4). The other 1556 DEGs were also categorized into the corresponding GO terms, but did not reach the significant level. These 56 DEGs were further categorized into three categories: biological process (34 DEGs), molecular function (14 DEGs), and cellular component (8 DEGs) (figure 8). In the cellular component category, 2 DEGs were significantly enriched in the neuromuscular junction class (*p* = 0.0173). In the biological process, 2 DEGs were enriched in the synapse assembly class (*p* = 0.0037); and in molecular function classes, 2 DEGs were enriched in the sulfiredoxin activity class (*p* = 0.002). All the GO analysis information is arranged in electronic supplementary material, table S4.

To further identify the active biochemical pathways of unigenes, genes were mapped to the KEGG database, which can be used to systematically analyse the functions and network of genes. Among them, a total of 12 976 genes (containing 137 DEGs) were annotated to 86 pathways in KEGG database, but no significance (*p *> 0.05). KEGG enrichment analysis found 24 and 11 differentially expressed genes involved in development and reproduction associated pathways, respectively. For example, laminin alpha 1 (*LAMA1*) is essential to form and maintain muscle integrity [40]. For Bardet–Biedl syndrome 10 (*BBS10*), its mutation could cause Bardet–Biedl syndrome, which includes diabetes and obesity [41]. The paternally expressed 10 gene (*PEG10*) plays important roles in cell proliferation, apoptosis and meat quality traits [42,43]. Histone deacetylase 9 (*HDAC9*) can promote cell proliferation, regulate cell cycle progression and inhibit apoptosis [44,45]. These results are listed in table 1, and all the pathways and related information are listed in electronic supplementary material, table S5. Although no significance was found in KEGG enrichment analysis, the KEGG analysed results were a good indication of the function of the genes which were enriched in specific pathways [29,37]. The above four genes, *LAMA1*, *BBS10*, *PEG10* and *HDAC9*, were all enriched in the right pathways, which was consistent with their functions (electronic supplementary material, table S5). This means that the KEGG analysis results could be used as reference to search the functions of new genes and reveal important genes.

**Table 1.** Differentially expressed genes involved in development and reproduction associated pathways.

no.	function^a^	development associated pathways	unigenes^b^
1	development	apoptosis	172(1)
2	development	regulation of actin cytoskeleton	352(3)
3	development	amyotrophic lateral sclerosis (ALS)	35(1)
4	development	MAPK signalling pathway, yeast	20(1)
5	development	Jak-STAT signalling pathway	159(3)
6	development	inositol phosphate metabolism	152(2)
7	development	ubiquitin mediated proteolysis	116(1)
8	development	MAPK signalling pathway	426(5)
9	development	hypertrophic cardiomyopathy (HCM)	181(1)
10	development	mTOR signalling pathway	119(2)
11	development	cardiac muscle contraction	187(2)
12	development	insulin signalling pathway	181(1)
13	development	Wnt signalling pathway	221(1)
14	reproduction	steroid hormone biosynthesis	13(1)
15	reproduction	endometrial cancer	127(2)
16	reproduction	ErbB signalling pathway	200(2)
17	reproduction	aldosterone-regulated sodium reabsorption	56(1)
18	reproduction	oocyte meiosis	230(1)
19	reproduction	progesterone-mediated oocyte maturation	117(2)
20	reproduction	GnRH signalling pathway	199(2)

^a^The functions of the pathways are development or reproduction associated.

^b^Refers to the numbers of total unigenes and differently expressed unigenes (in parentheses) mapped to the specific pathways.

### 3.4. Quantitative real-time polymerase chain reaction validation of the candidate differently expressed genes

**Figure. 9.**
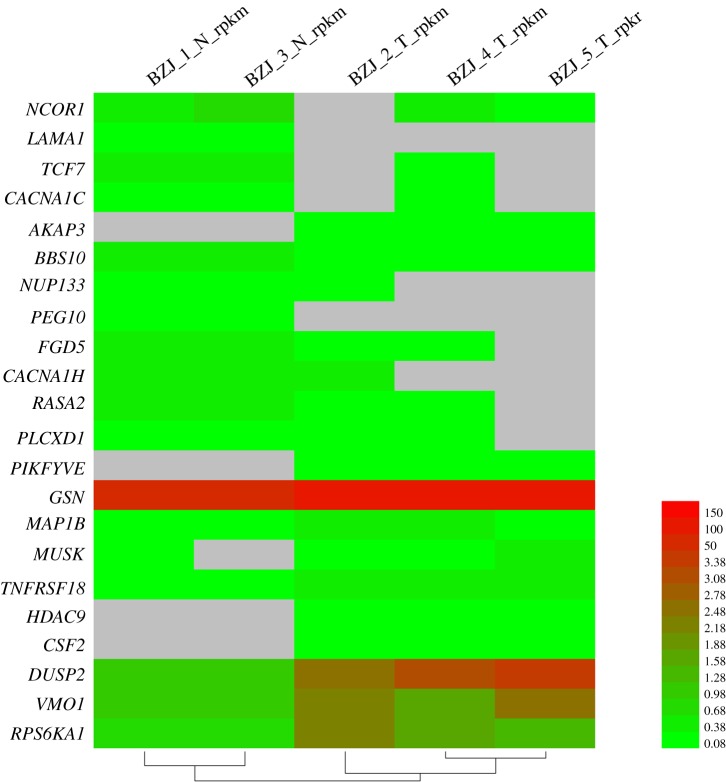
A heat-map exploring the differences in qRT-PCR detected genes expression between samples. Different colours represent different expression levels; the grey colour represents no expression. Control group: BZJ_1_N and BZJ_3_N; treatment group: BZJ_2_T, BZJ_4_T and BZJ_5_T.

**Figure 10.**
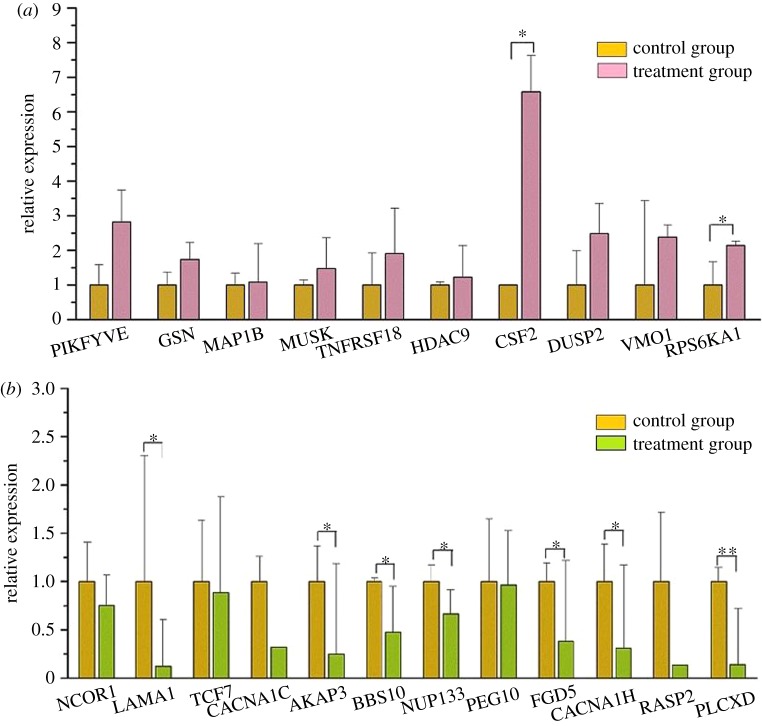
Quantification of the mRNA levels of differently expressed genes. The error bars are the s.e. of duplication. * and ** represent the significantly different expression at 0.05 and 0.01 level, respectively. (*a*) Upregulated genes and (*b*) downregulated genes.

To validate the reliability of the RNA-seq analysis, 22 candidate DEGs from different GO terms and KEGG pathways were successfully selected to perform qRT-PCR. A heat-map was produced to explore the difference expression of these 22 genes based on the RPKM values in RNA-seq (figure 9). The candidate DEG sequences were blasted to the reference RNA sequences in the National Center for Biotechnology Information (NCBI) website. The result of qRT-PCR showed that the expression changes of these 22 candidate DEGs in normal ewes and ovariectomized ewes longissimus dorsi muscle samples had the same trend as the results of RNA-seq. And among these 22 candidate DEGs, 9 and 1 genes were significantly and approximately significantly expressed in control and treatment group, respectively (figure 10). In addition, there were two upregulated and seven downregulated genes significantly differently expressed in ovariectomized group (figure 10). Considering the differences of the experimental methods, data calculation methods and statistical model, differences of expression levels within a certain range are inevitable and acceptable. Thus these results suggested that the result of RNA-seq is believable in this study.

Among the 22 validated unigenes, 20 and 8 genes were mapped to GO and KEGG databases, respectively. For example, microtubule associated protein 1B (*MAP1B*) gene, which is involved in microtubule assembly and neurogenesis [46,47], was categorized into microtubule bundle formation, microtubule associated complex, microtubule binding etc. GO terms. Calcium channel, voltage-dependent, L type, alpha 1C subunit (*CACNA1C*) gene encodes an important subunit in calcium channels, which is related to heart disease and psychosis [48]. In our study, this gene was categorized into arrhythmogenic right ventricular cardiomyopathy, calcium signalling pathway, cardiac muscle contraction, Alzheimer's disease etc. pathways. Thus KEGG and GO term analysis could well predict the possible function of unigenes and revealed potential genes that play crucial roles in this study.

## 4. Conclusion

In conclusion, we revealed 1612 DEGs in ovariectomized goat muscle, and 24 and 11 DEGs involved in development and reproduction associated pathways, respectively. These results provide valuable theoretical basis for the research of molecular mechanisms of female animal fattening and muscle development of ovariectomized animals.

## Supplementary Material

Fupplementary file: The list of the primers which were used for the qPCR analysis of 22 differently expressed genes

## Supplementary Material

Fupplementary file: Statistics data (including the data volume of raw data and valid data, and the valid ration of reads) of the sequencing result of five samples

## Supplementary Material

Fupplementary file: The list of the 1612 differently expressed genes

## Supplementary Material

Fupplementary file: The list of the genes which were mapped to Go terms

## Supplementary Material

Fupplementary file: The list of the genes which were enriched to specific KEGG pathway

## Data accessibility

The following information was supplied regarding the deposition of related data in NCBI experimental studies and data sets: Gene Expression Omnibus (GEO): GSE84110: http://www.ncbi.nlm.nih.gov/geo/query/acc.cgi?acc=GSE84110.
