# Supplementary material for: The muscle development transcriptome landscape of ovariectomized goat
Source: R Soc Open Sci. 2017 Dec 20;4(12):171415. doi: 10.1098/rsos.171415 (PMC5750031; doi:10.1098/rsos.171415)
Supplement: Fupplementary file: The list of the primers which were used for the qPCR analysis of 22 differently expressed genes [file rsos171415supp1.doc]

**Table S1** Primers used for the qPCR analysis of differently expressed genes

| Transcript ID number | Mapped gene | Primer sequence (5′–3′) | Product length (bp) | Mapped gene GenBank accession number |
| --- | --- | --- | --- | --- |
| comp48678_c4_seq59 | NCOR1 | F: TGCCCTCGCTGCTCTTGT | 86 | XM_012154411.2 |
|  |  | R: TGGTGGCGTCGTGCTTACT |  |  |
| comp137217_c0_seq1 | LAMA1 | F: AAGACTCTCCGGGCTACTGT | 250 | [XM_013974410.1](http://www.ncbi.nlm.nih.gov/nucleotide/926722974?report=genbank&log$=nucltop&blast_rank=1&RID=G1CD4AT9014) |
|  |  | R: CTGCTAGGAGCCCTTGCTTT |  |  |
| comp101922_c0_seq1 | PIKFYVE | F: GCTGGGAAATCTCCTGCTC | 155 | [XM_013971383.1](http://www.ncbi.nlm.nih.gov/nucleotide/926685570?report=genbank&log$=nucltop&blast_rank=25&RID=FTGSZ80G01R) |
|  |  | R: TTGCGTTCATCCTCAGTGGTC |  |  |
| comp85540_c0_seq1 | TCF7 | F: GGGAAGAGCAGGCCAAGTAT | 119 | [NM_001099186.2](http://www.ncbi.nlm.nih.gov/nucleotide/402746535?report=genbank&log$=nucltop&blast_rank=12&RID=G1CGHTRB014) |
|  |  | R: TTCCCTCGACCGTCTCTTCT |  |  |
| comp128033_c0_seq1 | CACNA1C | F: TTCACCCCAACGCTTACCTC | 140 | [XM_013964320.1](http://www.ncbi.nlm.nih.gov/nucleotide/926693023?report=genbank&log$=nucltop&blast_rank=2&RID=FTGTEB3F01R) |
|  |  | R:GAATCCAGCACCCTTCCCTC |  |  |
| comp132221_c0_seq1 | AKAP3 | F: GCACCTCCAACCTGTATCCC | 221 | [XM_013964240.1](http://www.ncbi.nlm.nih.gov/nucleotide/926692811?report=genbank&log$=nucltop&blast_rank=1&RID=G1C9VK6F014) |
|  |  | R: GGGTCTTTCATACTGGGGGC |  |  |
| comp56635_c0_seq1 | BBS10 | F: GGCGGAAGTGCTAGAAACCA | 186 | [XM_005679740.2](http://www.ncbi.nlm.nih.gov/nucleotide/926691029?report=genbank&log$=nucltop&blast_rank=3&RID=FTGTX4AT015) |
|  |  | R: AAGGATGCTCTAACGGGCTG |  |  |
| comp48182_c0_seq2 | GSN | F: CCAACCTTTACGGAGACTTCTTCACA | 275 | [NM_001113284.1](http://www.ncbi.nlm.nih.gov/nucleotide/164452942?report=genbank&log$=nucltop&blast_rank=1&RID=G1CMVP72015) |
|  |  | R: TGATGCCACGCCTCCTTTCTT |  |  |
| comp69579_c0_seq1 | MAP1B | F: AGACCCATGAGAAACAGCAGG | 122 | [NM_001206119.1](http://www.ncbi.nlm.nih.gov/nucleotide/329663570?report=genbank&log$=nucltop&blast_rank=11&RID=FTJ216MJ014) |
|  |  | R: TGCGGCTGACCTTGGTTGTT |  |  |
| comp111552_c0_seq1 | NUP133 | F: CGAGGAACACCTACGCGAATA | 176 | [NM_001076872.2](http://www.ncbi.nlm.nih.gov/nucleotide/402767292?report=genbank&log$=nucltop&blast_rank=7&RID=FTH2F5S901R) |
|  |  | R: CAGACCAGACAAGCCCATCC |  |  |
| comp120597_c0_seq1 | PEG10 | F: TCGGTTGGTGTGTGTCGAAG | 206 | [NM_001291299.1](http://www.ncbi.nlm.nih.gov/nucleotide/604723344?report=genbank&log$=nucltop&blast_rank=1&RID=FTH3241E01R) |
|  |  | R: GCTTTCTGTTTCACGCGAGG |  |  |
| comp88751_c0_seq1 | MUSK | F: TAACCCTCACTACTCTTCCTTC | 232 | [XM_013966264.1](http://www.ncbi.nlm.nih.gov/nucleotide/926700132?report=genbank&log$=nucltop&blast_rank=3&RID=FTH3J80G01R) |
|  |  | R: AGCATCTTCACTGCCACC |  |  |
| comp17413_c0_seq1 | FGD5 | F: AGCTTCCCGTTGCTGCATTT | 172 | [XM_012114236.1](http://www.ncbi.nlm.nih.gov/nucleotide/803218555?report=genbank&log$=nucltop&blast_rank=3&RID=FTH40ADS01R) |
|  |  | R: CCAAATTCTGCCCGTCGTCA |  |  |
| comp70723_c0_seq1 | CACNA1H | F: TCCTGCTCATCGTCAGCTTC | 178 | [XM_012144304.2](http://www.ncbi.nlm.nih.gov/nucleotide/965859656?report=genbank&log$=nucltop&blast_rank=1&RID=FTHBM6BJ015) |
|  |  | R: TAGTCCGCATAGTAGGGCCG |  |  |
| comp33223_c0_seq1 | TNFRSF18 | F: CTTAGCTGGGTCACGGTCAG | 147 | [XM_012138556.2](http://www.ncbi.nlm.nih.gov/nucleotide/965847638?report=genbank&log$=nucltop&blast_rank=1&RID=FTHEHCH6014) |
|  |  | R: TGGGTTTCCCACCCTGTTTC |  |  |
| comp118322_c0_seq1 | HDAC9 | F: CACAAATGCACTGGTGTTTCAG | 131 | [XM_013963175.1](http://www.ncbi.nlm.nih.gov/nucleotide/926689747?report=genbank&log$=nucltop&blast_rank=1&RID=G1CUUU0501R) |
|  |  | R: GCTCTCCAAGACCCACTCCTC |  |  |
| comp79695_c0_seq1 | CSF2 | F: CATTCCCTTTGACTGCTGG | 116 | [XM_013965265.1](http://www.ncbi.nlm.nih.gov/nucleotide/926695712?report=genbank&log$=nucltop&blast_rank=1&RID=FTHCGF27014) |
|  |  | R: TGTTTGGCTCTTTGTGGG |  |  |
| comp12302_c0_seq1 | DUSP2 | F: TAACTTTCCAGCGTCAACACAG | 240 | [XM_015093902.1](http://www.ncbi.nlm.nih.gov/nucleotide/965923624?report=genbank&log$=nucltop&blast_rank=1&RID=FTHCZHD601R) |
|  |  | R: TGGACTCTACACCCTCGACT |  |  |
| comp12478_c0_seq1 | RASA2 | F: AGCAGCCAGGTAGCAAAGATG | 245 | [XM_012102310.1](http://www.ncbi.nlm.nih.gov/nucleotide/803005408?report=genbank&log$=nucltop&blast_rank=2&RID=FTHJVZ1N01R) |
|  |  | R: TCCGTTTAGATACGCGGAGG |  |  |
| comp21217_c0_seq1 | VMO1 | F: CCAAGATCCAGCAGCCTCAA | 132 | [XM_005693465.2](http://www.ncbi.nlm.nih.gov/nucleotide/926716334?report=genbank&log$=nucltop&blast_rank=1&RID=FTHT220V014) |
|  |  | R: GCTTTAATAGCGGAGGTGGGA |  |  |
| comp19927_c0_seq1 | RPS6KA1 | F: TTGGCTCCTTTTCTGTTCTGT | 144 | [XM_005676810.2](http://www.ncbi.nlm.nih.gov/nucleotide/926686335?report=genbank&log$=nucltop&blast_rank=1&RID=FTHKNNB901R) |
|  |  | R: GGACTGCTCGATCTGACTGC |  |  |
| comp71922_c0_seq1 | PLCXD1 | F: GTCGCTGGTGTCTACGGTG | 103 | [NW_005101198.1](http://www.ncbi.nlm.nih.gov/nucleotide/541128341?report=genbank&log$=nucltop&blast_rank=1&RID=FTJ8BP29015) |
|  |  | R: CTCAGCGTGTCTTCCTGTGT |  |  |
| Internal reference | RPL19 | F: GGGTACTGCCAATGCTCGAA | 119 | XM_005693740 |
|  |  | R: TGTGATACATGTGGCGGTCA |  |  |
