# Supplementary material for: The muscle development transcriptome landscape of ovariectomized goat
Source: R Soc Open Sci. 2017 Dec 20;4(12):171415. doi: 10.1098/rsos.171415 (PMC5750031; doi:10.1098/rsos.171415)
Supplement: Fupplementary file: Statistics data (including the data volume of raw data and valid data, and the valid ration of reads) of the sequencing result of five samples [file rsos171415supp2.doc]

**Table S2** Statistics data of sequencing result

| Animal group | Sample | Raw Data | |  | Valid Data | | Valid |
| --- | --- | --- | --- | --- | --- | --- | --- |
|  |  | Read | Base |  | Read | Base | Ratio(reads) % |
| Control | BZJ_1_N | 55727350 | 5572735000 |  | 55621438 | 5562143800 | 99.81 |
| Control | BZJ_3_N | 51765492 | 5176549200 |  | 51690018 | 5169001800 | 99.85 |
| Treatment | BZJ_2_T | 53630772 | 5363077200 |  | 53527082 | 5352708200 | 99.81 |
| Treatment | BZJ_4_T | 55141840 | 5514184000 |  | 55036314 | 5503631400 | 99.81 |
| Treatment | BZJ_5_T | 50018086 | 5001808600 |  | 49947344 | 4994734400 | 99.86 |
